# HSP90AB1 Is a Host Factor Required for Transmissible Gastroenteritis Virus Infection

**DOI:** 10.3390/ijms242115971

**Published:** 2023-11-04

**Authors:** Daili Song, Yujia Zhao, Ying Sun, Yixiao Liang, Rui Chen, Yiping Wen, Rui Wu, Qin Zhao, Senyan Du, Qigui Yan, Xinfeng Han, Sanjie Cao, Xiaobo Huang

**Affiliations:** 1 Research Center for Swine Diseases, College of Veterinary Medicine, Sichuan Agricultural University, Chengdu 611130, China; 2 Sichuan Science-Observation Experimental Station for Veterinary Drugs and Veterinary Diagnostic Technology, Ministry of Agriculture, Chengdu 611130, China; 3 National Animal Experiments Teaching Demonstration Center, Sichuan Agricultural University, Chengdu 611130, China

**Keywords:** TGEV, HSP90, viral infection, inhibitory effect, proinflammatory cytokines

## Abstract

Transmissible gastroenteritis virus (TGEV) is an important swine enteric coronavirus causing viral diarrhea in pigs of all ages. Currently, the development of antiviral agents targeting host proteins to combat viral infection has received great attention. The heat shock protein 90 (HSP90) is a critical host factor and has important regulatory effects on the infection of various viruses. However, its roles in porcine coronavirus infection remain unclear. In this study, the effect of HSP90 on TGEV infection was evaluated. In addition, the influence of its inhibitor VER-82576 on proinflammatory cytokine (IL-6, IL-12, TNF-α, CXCL10, and CXCL11) production induced by TGEV infection was further analyzed. The results showed that the knockdown of HSP90AB1 and HSP90 inhibitor VER-82576 treatment resulted in a reduction in TGEV M gene mRNA levels, the N protein level, and virus titers in a dose-dependent manner, while the knockdown of HSP90AA1 and KW-2478 treatment had no significant effect on TGEV infection. A time-of-addition assay indicated that the inhibitory effect of VER-82576 on TGEV infection mainly occurred at the early stage of viral replication. Moreover, the TGEV-induced upregulation of proinflammatory cytokine (IL-6, IL-12, TNF-α, CXCL10, and CXCL11) expression was significantly inhibited by VER-82576. In summary, these findings indicated that HSP90AB1 is a host factor enhancing TGEV infection, and the HSP90 inhibitor VER-82576 could reduce TGEV infection and proinflammatory cytokine production, providing a new perspective for TGEV antiviral drug target design.

## 1. Introduction

Transmissible gastroenteritis virus (TGEV) is a member of the genus Alphacoronavirus of the family Coronaviridae [1]. It is the main causative agent of transmissible gastroenteritis (TGE), which causes diarrhea, vomiting, and dehydration in pigs of various ages [2]. Specifically, the mortality rate of piglets less than two weeks old is close to 100% [3]. In addition, TGEV can infect various host cells, including PK15, ST, IPI-2I, and IPEC-J2 cells, in vitro [4,5]. The first occurrence of TGEV was reported in the United States in 1946 [6]. Subsequently, it was identified in South America, Asia, Africa, and Europe, causing massive economic losses in the global pig breeding industry [7,8]. Currently, the major commercially available TGEV vaccines utilize live-attenuated viral strains, including PROSYSTEM^®^ TGE/Rota and PROSYSTEM^®^ TREC [9]. These vaccines can promote antibody production in sows and confer lactogenic immunity to piglets, thereby transiently improving piglet resistance [10]. However, the protective effects are transient, as maternally derived antibody titers wane with age [2]. Therefore, screening and identifying effective antiviral agents for TGEV is of great significance for TGEV prevention and treatment.

It is well known that viruses rely on host factors to complete their infection, and some host factors play important roles in viral entry, replication, assembly, and release [11]. Recently, the host factors involved in TGEV infection have been reported. For example, transferrin receptor 1 (TRF1) is a supplementary receptor that assists TGEV entry into porcine intestinal epithelium [12]. Transmembrane protein 41B (TMEM41B) is a host factor required for TGEV replication and contributes to the formation of coronavirus replication organelles [13]. Eukaryotic translation initiation factor 4-alpha (EIF4A2) and UBX domain-containing protein 1 (UBXN1) play roles in viral replication and can interact with the membrane and spike protein of TGEV, respectively [14,15]. Moreover, increasing studies have indicated that small molecule inhibitors can inhibit TGEV replication by regulating intracellular signaling pathways. For example, receptor tyrosine kinase inhibitor A9 and Na+/K+-ATPase inhibitor ouabain block TGEV replication via p38 MAPK [16] and JAK1 or PI3K/PDK1 [17,18] signaling pathways. In addition, interferon γ [19] and interferon α [20,21] have been suggested to inhibit TGEV replication in vivo and in vitro.

Heat shock proteins (HSPs) are highly conserved molecular chaperone proteins that play critical roles in cell signaling pathways, cell homeostasis, and survival [22]. HSP90 is a major member of the HSP family that can regulate the infection of various viruses [23]. Previous studies have reported that most inhibitors targeting HSP90 can block viral infection at the stages of viral entry [24], viral assembly [25], and viral gene expression [26]. To date, the function of HSP90 on human coronavirus has been evaluated by several groups. For example, Li et al. [27] found that HSP90 inhibitor 17-AAG could significantly inhibit Middle East respiratory syndrome coronavirus (MERS-CoV), severe acute respiratory syndrome coronavirus 2 (SARS-CoV-2), and SARS-CoV replication. Further research has revealed that HSP90AB1 could regulate SARS-CoV-2 infection via binding viral RNA [28]. Transcriptomic analysis identified HSP90AA1 as a host factor relevant for SARS-CoV-2 infection in human cell lines [29]. In our previous study, HSP90AB1 was identified as a host factor that could potentially interact with porcine deltacoronavirus (PDCoV) by co-immunoprecipitation (Co-IP) coupled with LC/MS-MS [30]. Moreover, we also found that HSP90AB1 inhibitors 17-AAG and VER-82576 could reduce PDCoV infection. However, HSP90AA1 inhibitor KW-2478 had no significant inhibitory effect on PDCoV infection [31]. To date, there are few reports about the roles of HSP90 on TGEV infection.

In this study, the effect of HSP90 on TGEV infection was evaluated. Moreover, the production of the proinflammatory cytokines IL-6, IL-12, TNF-α, CXCL10, and CXCL11 induced by TGEV and the inhibitory effect of VER-82576 on the expression of these proinflammatory cytokines were evaluated. Our results revealed that HSP90AB1 knockdown and VER-82576 treatment reduced TGEV infection. Further research found that VER-82576 had a significant inhibitory effect on TGEV infection at the early stage of viral replication. Moreover, TGEV-induced mRNA levels of the proinflammatory cytokines IL-6, IL-12, TNF-α, CXCL10, and CXCL11 were significantly reduced by the addition of VER-82576.

## 2. Results

### 2.1. Viral Replication Kinetics of TGEV in ST Cells

**Figure 1:**
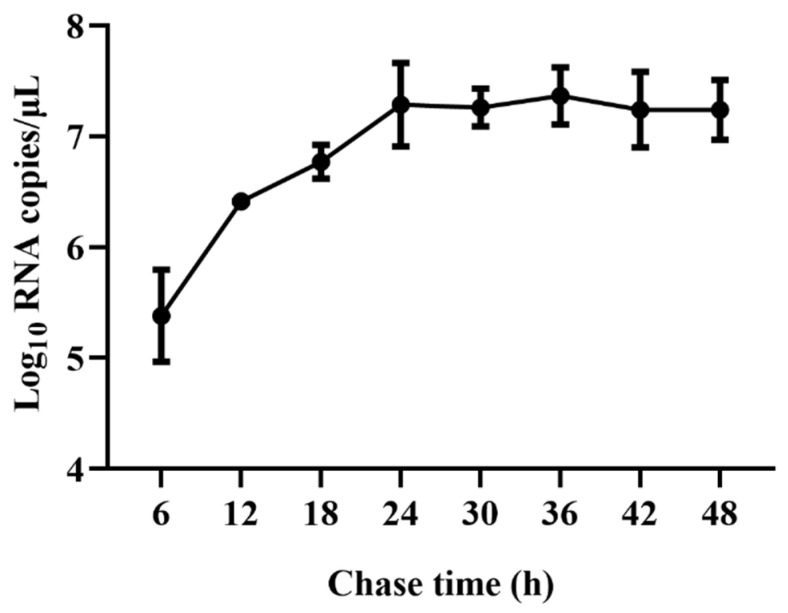
qRT-PCR analysis of viral replication kinetics of TGEV in ST cells. ST cells were infected with TGEV, and viral RNA copies were detected at 6, 12, 18, 24, 36, 42, and 48 hpi using qRT-PCR.

Viral RNA copies were detected at 6, 12, 18, 24, 36, 42, and 48 hpi to characterize the viral replication kinetics of TGEV in ST cells. The results showed that viral RNA copies rapidly increased from 6 hpi and reached a plateau at 24 hpi, with viral RNA copies of 7.28 Log_10_ copies/μL. No significant further increase from 24 to 48 hpi was observed (Figure 1). Therefore, approximately 24 hpi was used as an ending point of viral infection for the subsequent assays.

### 2.2. HSP90AB1 Enhances TGEV Infection

**Figure 2:**
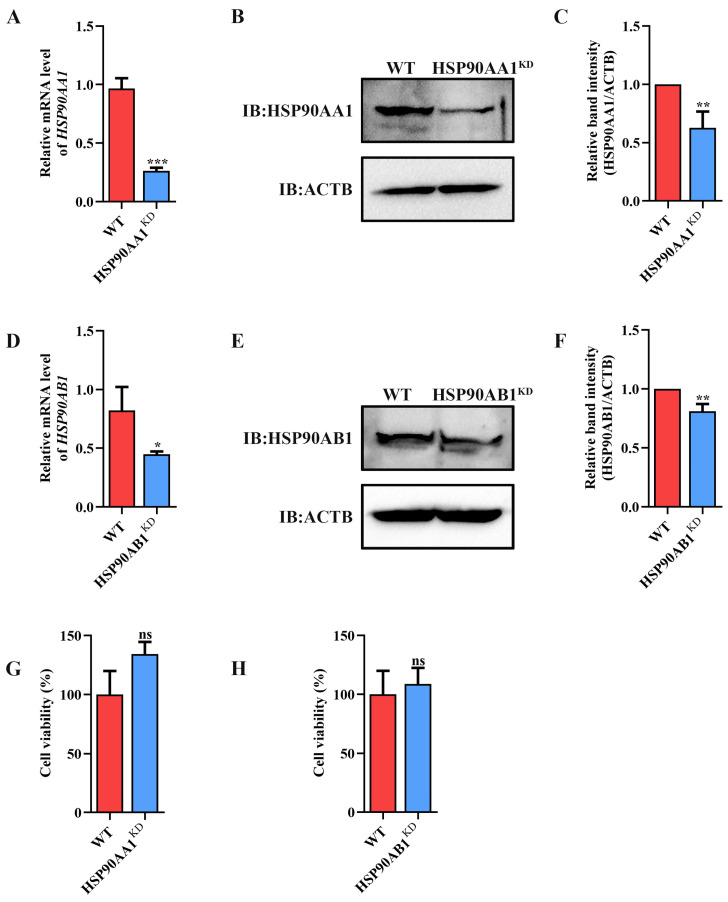
Identification of HSP90AA1 and HSP90AB1 knockdown cells. (**A**) qRT-PCR and Western blot of HSP90AA1 gene mRNA and (**B**) protein levels in WT and HSP90AA1 KD cells. (**C**) The band density of the protein was quantified using Image J software, and the HSP90AA1 to ACTB ratios were normalized to the control. (**D**) qRT-PCR and Western blot of HSP90AB1 gene mRNA and (**E**) protein levels in WT and HSP90AB1 KD cells. (**F**) The band density of the protein was quantified using Image J software, and the HSP90AB1 to ACTB ratios were normalized to the control. (**G**,**H**) Cell viability of WT and (**G**) HSP90AA1 or (**H**) HSP90AB1 KD cells were evaluated by CCK-8 assay. * means *p* ≤ 0.05, ** means *p* ≤ 0.01, *** means *p* ≤ 0.001, ns means *p* > 0.05.

**Figure 3:**
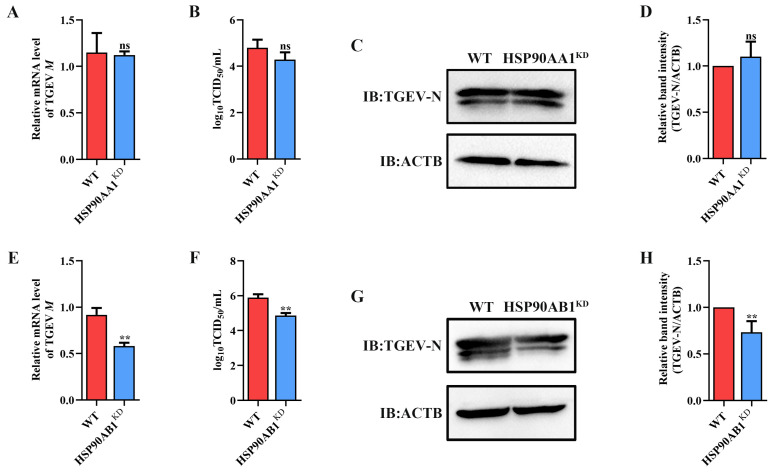
Effect of HSP90AA1 and HSP90AB1 knockdown on TGEV infection. (**A**) TGEV M gene mRNA levels, (**B**) viral titers in the cell supernatants, and (**C**) N protein levels in WT and HSP90AA1 KD cells. (**D**) The band density of the protein was quantified using Image J software, and the TGEV-N to ACTB ratios were normalized to the control. (**E**) TGEV M gene mRNA levels, (**F**) viral titers in the cell supernatants, and (**G**) N protein levels in WT and HSP90AB1 KD cells. (**H**) The band density of the protein was quantified using Image J software, and the TGEV-N to ACTB ratios were normalized to the control. ** means *p* ≤ 0.01, ns means *p* > 0.05.

**Figure 4:**
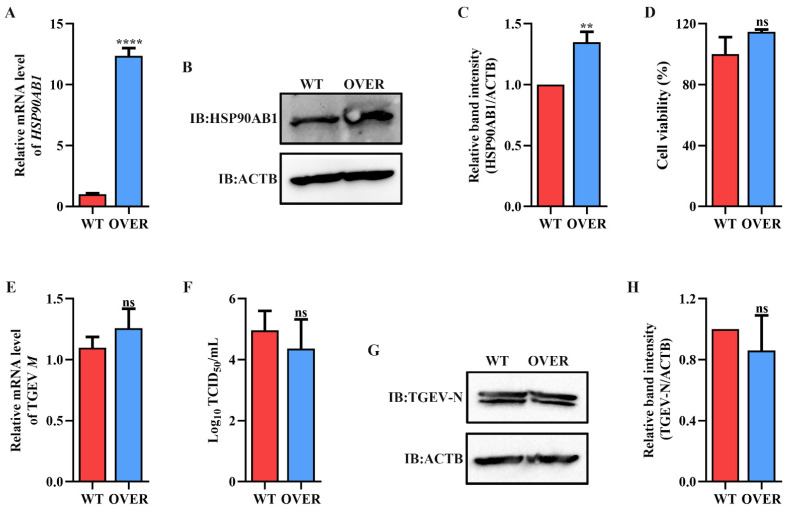
Effect of HSP90AB1 overexpression on TGEV infection. (**A**) qRT-PCR and Western blot of HSP90AB1 gene mRNA and (**B**) protein levels in WT and OVER cells. (**C**) The band density of the protein was quantified using Image J software, and the HSP90AB1 to ACTB ratios were normalized to the control. (**D**) Cell viability of WT and OVER cells was evaluated by CCK-8 assay. (**E**) TGEV M gene mRNA levels, (**F**) viral titers in the cell supernatants, and (**G**) N protein levels in WT and OVER cells. (**H**) The band density of the protein was quantified using Image J software, and the TGEV-N to ACTB ratios were normalized to the control. ** means *p* ≤ 0.01, **** means *p* ≤ 0.0001, ns means *p* > 0.05.

To determine the effect of HSP90 on TGEV infection, HSP90AA1 or HSP90AB1 knockdown cells were first constructed. qRT-PCR and Western blot results showed that the knockdown of HSP90AA1 resulted in about a 75% reduction in the HSP90AA1 gene mRNA level and a 37% decrease in the HSP90AA1 protein level (Figure 2A–C), while the HSP90AB1 gene mRNA and protein level were reduced by about 55% and 20% in the HSP90AB1 knockdown cells, respectively (Figure 2D–F). By CCK-8 assay, there was no significant difference in the viability of HSP90AA1 or HSP90AB1 knockdown cells compared to WT (Figure 2G,H). Subsequently, these cells were infected with TGEV. As shown in (Figure 3A–D), the knockdown of HSP90AA1 had no influence on the TGEV M gene mRNA level, viral titer, and N protein level. However, TGEV M gene mRNA levels were lower by 42% in HSP90AB1 knockdown cells (Figure 3E). Viral titers in the supernatant were also reduced by approximately 1 Log_10_ TCID_50_/mL (Figure 3F) and the N protein level was reduced by approximately 27% (Figure 3G,H), indicating that the knockdown of HSP90AB1 significantly reduces TGEV infection. Therefore, the effect of HSP90AB1 overexpression on TGEV infection was also evaluated, and the results found that HSP90AB1 overexpression led to a 12-fold and 1.4-fold increase in HSP90AB1 gene mRNA and protein level, respectively (Figure 4A–C). Interestingly, overexpression did not enhance viral replication (Figure 4D–H), and thus, this factor is likely necessary for infection, but not sufficient on its own to enhance it. 

### 2.3. HSP90 Inhibitor VER-82576 Could Inhibit TGEV Infection

**Figure 5:**
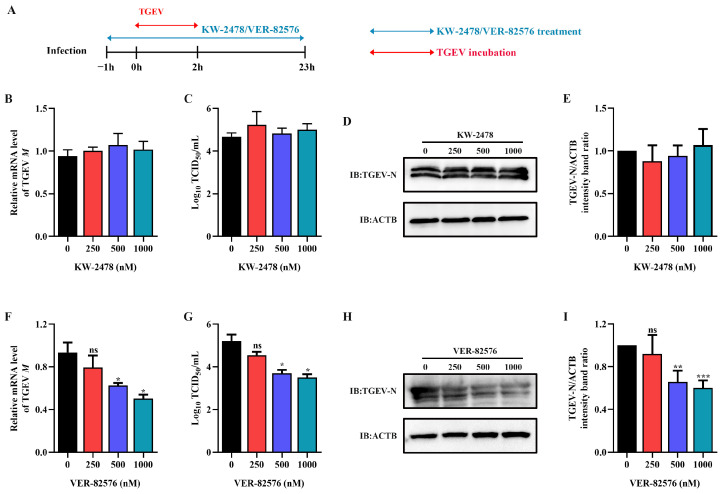
VER-82576 could inhibit TGEV infection. (**A**) Schematic diagram of ST cells treated with the HSP90 inhibitors VER-82576 and KW-2478. Blue double-headed arrows indicate KW-2478 or VER-82576 treatment, and red double-headed arrows indicate TGEV incubation. (**B**,**F**) The relative mRNA level of the TGEV M gene was determined by qRT-PCR. (**C**,**G**) Viral titer in the supernatant was analyzed by TCID_50_ and (**D**,**H**) N protein level was determined by Western blot. (**E**,**I**) The band density of the protein was quantified using Image J software, and the TGEV-N to ACTB ratios were normalized to the control. * means *p* ≤ 0.05, ** means *p* ≤ 0.01, *** means *p* ≤ 0.001, ns means *p* > 0.05.

To analyze the inhibitory effect of HSP90 inhibitors VER-82576 and KW-2478 on TGEV infection, ST cells were pretreated with 0, 250, 500, and 1000 nM VER-82576 or KW-2478 for 1 h and incubated with a virus in the presence of VER-82576 or KW-2478. Then, the viral supernatant was removed, and the cells were further cultured with maintenance medium containing inhibitors until 23 h after viral infection (Figure 5A). The results showed that the TGEV M gene mRNA level, N protein level, and viral titer were not significantly affected by the addition of KW-2478 as compared to the control (Figure 5B–E), suggesting that KW-2478 did not reduce TGEV infection. However, treatment with 500 nM and 1000 nM VER-82576 resulted in 39% and 50% reductions in TGEV M gene mRNA levels, respectively (Figure 5F). Moreover, VER-82576 treatment also resulted in a decrease in the viral titer and N protein level (Figure 5G–I). These results indicated that the HSP90 inhibitor VER-82576 had a significant inhibitory effect on TGEV infection in a dose-dependent manner.

### 2.4. TGEV Infection Had No Influence on HSP90 Expression

**Figure 6:**
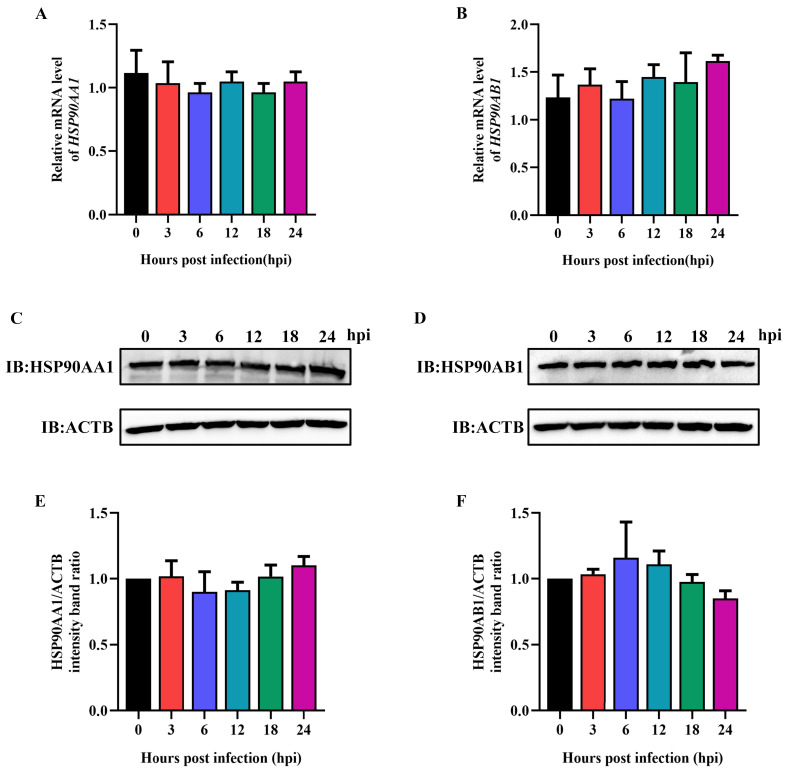
TGEV infection does not affect HSP90AA1 and HSP90AB1 expression. (**A**) HSP90AA1 or (**B**) HSP90AB1 gene mRNA level from ST cells infected with TGEV for 0, 3, 6, 12, 18, and 24 hpi were analyzed by qRT-PCR. (**C**) HSP90AA1 or (**D**) HSP90AB1 protein level from ST cells infected with TGEV for 0, 3, 6, 12, 18, and 24 hpi was analyzed by Western blot. (**E**,**F**) The band density of the protein was quantified using Image J software, and the (**E**) HSP90AA1 to ACTB and (**F**) HSP90AB1 to ACTB ratios were normalized to the control.

To verify the influence of TGEV infection on HSP90AA1 and HSP90AB1 expression, ST cells were infected with TGEV at an MOI of 0.1, then harvested over a 24 h time course. qRT-PCR and Western blot results showed that TGEV infection had no effect on HSP90AA1 and HSP90AB1 gene mRNA or protein levels (Figure 6), indicating that TGEV infection had no influence on HSP90 expression.

### 2.5. VER-82576 Inhibits TGEV Infection at the Early Stage of Replication

**Figure 7:**
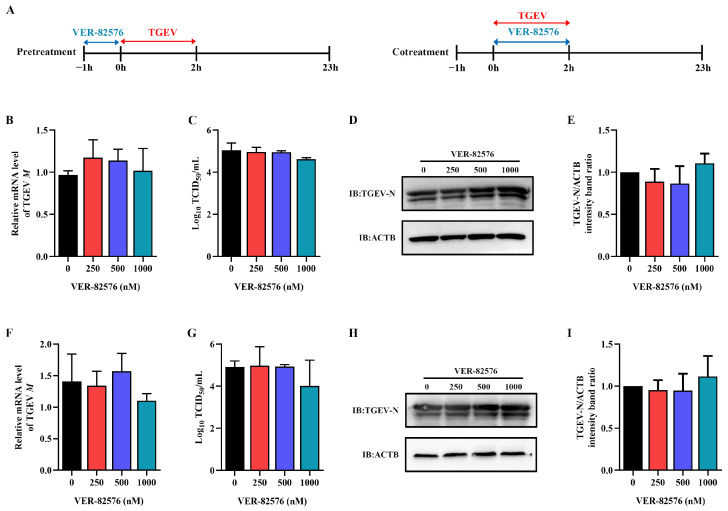
VER-82576 had no influence on the entry stage of TGEV infection. (**A**) Schematic diagram of ST cells treated with VER-82576 before (−1–0 h) or during (0–2 h) TGEV incubation. Blue double-headed arrows indicate VER-82576 treatment, and red double-headed arrows indicate TGEV incubation. (**B**,**F**) The relative mRNA level of TGEV M gene was determined by qRT-PCR. (**C**,**G**) Viral titer in the supernatant was analyzed by TCID_50_, and (**D**,**H**) N protein level was determined by Western blot. (**E**,**I**) The band density of the protein was quantified using Image J software, and the TGEV-N to ACTB ratios were normalized to the control.

To further detect the key stage of the viral infection cycle affected by VER-82576, ST cells were treated with 0, 250, 500, and 1000 nM VER-82576 for 1 h prior to TGEV infection (−1–0 h) or 2 h during TGEV incubation (0–2 h). The viral supernatant was removed, and the cells were further cultured with maintenance medium without VER-82576 until 23 h after viral infection (Figure 7A). qRT-PCR, TCID_50_, and Western blot results showed that VER-82576 had no inhibitory effect on the TGEV M gene mRNA level, virus titer, and N protein level when it was added before (Figure 7B–E) or during TGEV incubation (Figure 7F–I), indicating that VER-82576 inhibited TGEV infection mainly at the post-entry stage of viral infection.

**Figure 8:**
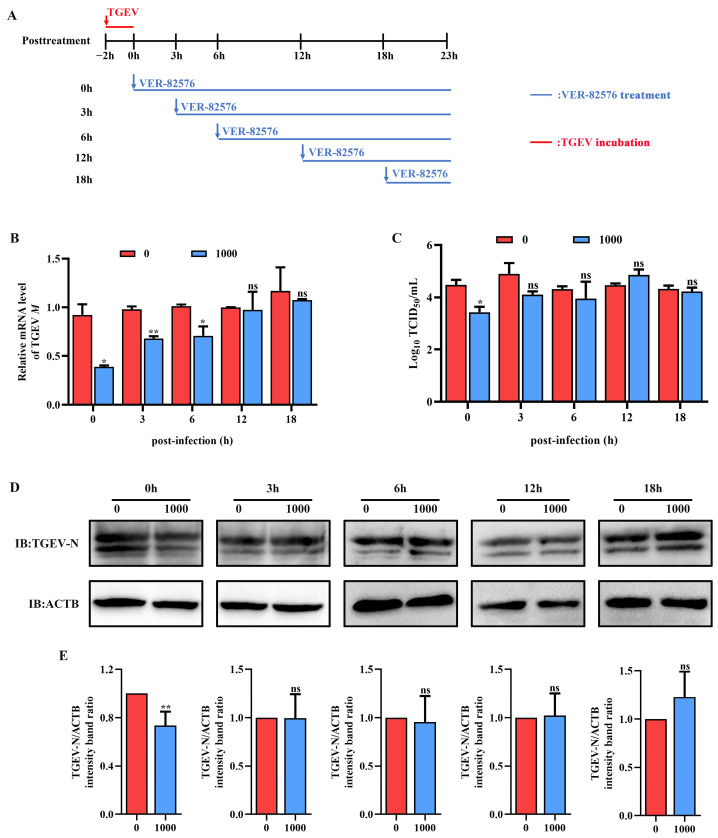
VER-82576 inhibits TGEV infection at the early stage of viral replication. (**A**) Schematic diagram of ST cells treated with VER-82576 at different time points after viral entry. Blue arrows indicate the time point at which VER-82576 treatment began, and the blue line indicates the presence of VER-82576. Red down arrows and the red line indicate TGEV incubation. (**B**) TGEV M gene mRNA levels, (**C**) viral titers in the cell supernatants, and (**D**) N protein levels in VER-82576 treated ST cells. (**E**) The band density of the protein was quantified using Image J software, and the TGEV-N to ACTB ratios were normalized to the control. * means *p* ≤ 0.05, ** means *p* ≤ 0.01, ns means *p* > 0.05.

To confirm that VER-82576 mainly inhibits TGEV infection in the post-entry stages, we replaced the culture medium containing 1000 nM VER-82576 at different time points after viral infection (0, 3, 6, 12, 18 hpi), using untreated cells as the control. The samples were collected uniformly at 23 h post-infection for analysis (Figure 8A). The results showed that the addition of VER-82576 at 0 and 3 hpi resulted in 61% and 32% reductions of the TGEV M gene mRNA levels, respectively, and a 1 Log_10_ TCID_50_/mL decrease was also observed at the viral titer level at 0 hpi. In addition, the TGEV M gene mRNA level was downregulated by 30%, while the viral titer in the supernatant was not significantly different when VER-82576 was added at 6 hpi. VER-82576 treatment at 12 and 18 hpi had no notable inhibitory effect on the TGEV M gene mRNA levels or viral titers (Figure 8B,C). N protein levels were lower by 25% in VER-82576 treated cells at 0 hpi and had no significant difference at 3, 6, 12, and 18 hpi (Figure 8D,E). These findings indicated that VER-82576 could inhibit TGEV infection at the early stage of viral replication.

### 2.6. Effect of VER-82576 on TGEV-Induced Proinflammatory Cytokine Production

**Figure 9:**
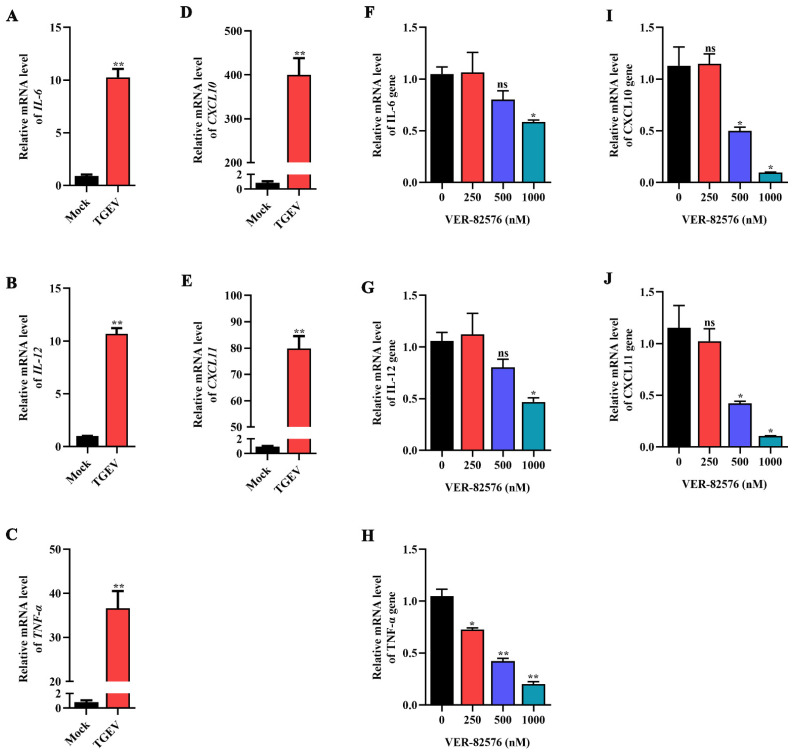
VER-82576 inhibited the production of proinflammatory cytokines induced by TGEV infection. (**A**–**E**) qRT-PCR detection of the mRNA levels of proinflammatory cytokines: (**A**) IL-6, (**B**) IL-12, (**C**) TNF-α, (**D**) CXCL10, and (**E**) CXCL11 induced by TGEV infection. (**F**–**J**) qRT-PCR detection of the inhibitory effect of VER-82576 on the mRNA level of proinflammatory cytokines: (**F**) IL-6, (**G**) IL-12, (**H**) TNF-α, (**I**) CXCL10, and (**J**) CXCL11 induced by TGEV infection. * means *p* ≤ 0.05, ** means *p* ≤ 0.01, ns means *p* > 0.05.

To verify the effect of TGEV infection on some representative proinflammatory cytokines, the mRNA levels of IL-6, IL-12, TNF-α, CXCL10, and CXCL11 were analyzed by qRT-PCR. The results indicated that these proinflammatory cytokines were significantly increased upon TGEV infection, among which CXCL10 was the explosively upregulated proinflammatory cytokine, increasing approximately 400-fold in ST cells (Figure 9A–E), indicating that TGEV infection could induce the production of proinflammatory cytokines.

ST cells were treated with 0, 250, 500, and 1000 nM VER-82576, and the inhibitory effect of VER-82576 on the production of TGEV-induced proinflammatory cytokines was detected by qRT-PCR. Treatment with 250 nM VER-82576 resulted in the mRNA level of TNF-α being downregulated by about 28%, but had no significant effect on IL-6, IL-12, CXCL10, and CXCL11 mRNA levels. In addition, 500 nM VER-82576 treatment resulted in an approximate 50% decrease in TNF-α, CXCL10, and CXCL11 mRNA levels without an obvious reduction in IL-6 and IL-12 mRNA levels. The levels of IL-6, IL-12, TNF-α, CXCL10, and CXCL11 were downregulated by about 42%, 53%, 80%, 91%, and 90%, respectively, by the addition of 1000 nM VER-82576 (Figure 9F–J). These results indicated that VER-82576 could dampen the induction of proinflammatory cytokine production induced by TGEV infection.

## 3. Discussion

TGEV is an important enteropathogen that causes diarrhea, vomiting, and dehydration in pigs of various ages. To date, host factors, including porcine aminopeptidase N (APN) [32], TRF1 [12], TMEM41B [13], EIF4A2 [14,15], and UBXN1 [14,15], play a positive role in TGEV infection. Moreover, some host protein inhibitors, including the receptor tyrosine kinase inhibitor A9 [16] and the Na+/K+-ATPase inhibitor ouabain [17], can inhibit TGEV infection to various extents.

Regarding the in vitro culture of TGEV, ST cells are widely used for viral isolation and propagation [4]. In this study, the replication kinetics of the TGEV strain SC-H in ST cells were determined, and qRT-PCR results showed that viral RNA copies reached a plateau at 24 hpi (Figure 1), indicating that 24 hpi was the right time to collect samples for evaluating the effect of antiviral drugs on TGEV. Ma et al. [33] pointed out that TGEV infection resulted in an increase in HSPA1B and HSP60 protein expression levels. Here, we found that TGEV infection had no effect on HSP90AA1 and HSP90AB1 expression at any post-infection time point tested (Figure 6). These results are consistent with Ma R et al. [33] who found that HSP90AA1 and HSP90AB1 were unchanged in TGEV-infected ST cells at 48 hpi. Interestingly, these two proteins were significantly downregulated at 64 hpi, which may be attributed to the host’s immune responses or a decrease in infected cell viability as viral infection progressed. 

Currently, antiviral drugs mainly include directly acting antivirals (DAAs) and host-acting antivirals (HAAs) [34]. Due to the advantages of reducing viral drug resistance, the development of HAAs that can combat viral infection has received great attention [35]. HSP90, a critical host factor, can regulate a variety of viral infections at multiple stages of the viral life cycle [23]. In mammals, there are four isoforms of HSP90: HSP90AA1, HSP90AB1, glucose-regulated protein 94 (GRP94), and tumor necrosis factor receptor-associated protein 1 (TRAP1) [23,36]. Some studies have reported that HSP90 inhibitors can bind to different HSP90 isoforms and inhibit their ATPase activity. For example, AUY-922 can act on HSP90AA1 and HSP90AB1, but has weak effects on GRP94 or TRAP1 [37]. The HSP90 inhibitor KW-2478 has a high affinity for HSP90AA1, while VER-82567 as a pan-HSP90 inhibitor has strong inhibitory activity and selectivity towards HSP90AB1 [38,39,40]. Hence, in this study, KW-2478 and VER-82576 were chosen to evaluate their inhibitory effect on TGEV infection. Our results showed that KW-2478 did not significantly affect TGEV infection, while VER-82576 inhibited TGEV infection in a dose-dependent manner (Figure 5), indicating that VER-82576 has strong selectivity in inhibiting the HSP90AB1 and HSP90AB1 ATPase activity that might be required for TGEV infection. We also found that HSP90AB1 knockdown could reduce TGEV infection, whereas HSP90AA1 knockdown had no influence on TGEV infection (Figure 3), suggesting that it is HSP90AB1, not HSP90AA1, that could function as a critical host factor for TGEV infection. In addition, previous research showed that HSP90 could block various viral infections by interfering with viral entry [24], genome replication [41], nuclear egress [25], assembly [25,42], protein expression [43], and other processes. In this study, our results revealed that VER-82576 treatment at 0 and 3 hpi resulted in 61% and 32% reductions of TGEV M gene mRNA levels, a 25% reduction of the TGEV N protein, and a 1 Log10 TCID50/mL decrease in the viral titer level at 0 hpi (Figure 8), while it had no influence on the TGEV M gene mRNA level and viral titer when added before TGEV infection (−1–0 h) or during TGEV incubation (0–2 h) (Figure 7), indicating that VER-82576 inhibits TGEV infection mainly at the early stage of viral replication.

TGEV infection resulted in the excessive induction of proinflammatory cytokines, which indicated that the inflammatory response is strongly associated with TGEV pathogenesis. Xia et al. [44] showed that TGEV infection significantly upregulated the expression of proinflammatory cytokines (IL-1β, IL-6, and TNF-α) and anti-inflammatory cytokines (IL-10 and TGF-β) in the jejunum tissue of piglets. In vitro experiments showed that TGEV infection can also induce the production of cytokines IL-1β, IL-6, IL-8, TGF-β, TNF-α, CCL2, CCL5, CXCL16, and RANTES [33,45]. However, Zhao et al. [46] found that the production of IL-12, IFN-γ, and IL-10 in virulent SHXB-infected immature monocyte-derived dendritic cells (Mo-DCs) was significantly lower than that in UV-inactivated SHXB-stimulated cells. In addition, the levels of proinflammatory cytokines IL-1β, IL-6, IL-8, and TNF-α in enterotoxigenic Escherichia coli K88 (ETEC K88)- and TGEV-coinfected cells were higher than those in TGEV-infected cells alone and higher than those in cells infected with ETEC K88 alone [47]. In this study, some representative proinflammatory cytokines (IL-6, IL-12, TNF-α, CXCL11, and CXCL10) were significantly induced by the TGEV infection of ST cells (Figure 9A–E), among which CXCL10 was an explosively upregulated proinflammatory cytokine with approximately 400-fold upregulation (Figure 9E), which further confirmed that the inflammatory response is closely related to TGEV pathogenesis.

The nuclear factor kappa B (NF-κB) signaling pathway is one of the most important signaling pathways involved in regulating proinflammatory cytokine production [48], and HSP90 plays a critical role in the activation of NF-κB [49]. Previous research indicated that 17-DMAG could reduce the upregulation of proinflammatory cytokines (IL-10 and TNF-α) during porcine circovirus type 2 (PCV2) infection and that AT-533 could also reduce the TNF-α, IL-1β, and IL-6 production induced by herpes simplex virus type 1 (HSV-1) infection [50,51]. In addition, HSP90 inhibitors onalespib, ganetespib, and 17-AAG could reduce the SARS-CoV-2-induced production of IL-6, CXCL10, and CXCL11 [29]. Our results showed that 1000 nM VER-82576 caused the downregulation of IL-6, IL-12, TNF-α, CXCL11, and CXCL10 by about 42%, 53%, 80%, 90%, and 91%, respectively, induced by TGEV infection (Figure 9F–J), which suggested that proinflammatory cytokine expression levels could be used as an evaluation indicator for the TGEV treatment effect.

In conclusion, HSP90AB1 knockdown and VER-82576 treatment could reduce TGEV infection, and the inhibitory effect of VER-82576 on TGEV infection might occur at the post-entry stage of viral infection. Moreover, VER-82576 could also inhibit the TGEV-induced expression of proinflammatory cytokines (IL-6, IL-12, TNF-α, CXCL10, and CXCL11). Our findings will provide a new perspective for TGEV antiviral drug target design.

## 4. Materials and Methods

### 4.1. Cells, Virus, and Inhibitors

ST and HEK293T cells were maintained in Dulbecco’s modified Eagle’s medium (DMEM) (Gibco, Carlsbad, CA, USA) and supplemented with 10% fetal bovine serum (FBS) (PNA, Aidenbach, Germany) and 1% antibiotic-antimycotic (Solarbio, Beijing, China) at 37 °C in a humidified atmosphere of 5% CO2. The TGEV strain SC-H was isolated and stored by our laboratory. HSP90 inhibitors KW-2478 (HY-13468) and VER-82576 (HY-10942) were purchased from MedChemExpress (MedChemExpress, Monmouth Junction, NJ, USA). The anti-HSP90AB1 rabbit polyclonal antibody (11405-1-AP) and anti-HSP90AA1 rabbit polyclonal antibody (13171-1-AP) were purchased from the Proteintech Group (Chicago, IL, USA). The anti-TGEV N mouse monoclonal antibody was prepared by our laboratory. The HRP-conjugated goat anti-mouse IgG (AS003), HRP-conjugated goat anti-rabbit IgG (AS014), and anti-ACTB rabbit polyclonal antibody (AC026) were purchased from Abclonal (Wuhan, China).

### 4.2. Viral Replication Kinetics of TGEV in ST Cells

ST cells at 90% confluence in T75 flasks were washed three times with DMEM supplemented with 5 μg/mL of trypsin (henceforth referred to as maintenance media) and then inoculated with TGEV at a multiplicity of infection (MOI) of 0.1. The virus was allowed to adsorb for 2 h at 37 °C, and then 20 mL of the maintenance medium was added to each flask. Virus RNA copies in the supernatants collected at 6, 12, 18, 24, 36, 42, and 48 hpi were detected by qRT-PCR. 

### 4.3. Effect of the HSP90 on TGEV Infection

An sgRNA targeting HSP90AA1 or HSP90AB1 was synthesized and inserted into a lentiCRISPR v2 vector to generate HSP90AA1 and HSP90AB1 knockdown cells. The sgRNA sequences were shown in Table 1. The HSP90AB1 gene was also synthesized and inserted into a pCDH-CMV-MCS-EF1-copGFP-T2A-puro vector to construct HSP90AB1 overexpression cells. These plasmids were packaged into lentivirus particles in HEK293T cells by co-transfection with psPAX2 and pMD2.G at a ratio of 5:3:2, respectively. Subsequently, 48 h after transfection, the supernatants were collected and centrifuged 5000 rpm for 10 min at 4 °C. Lentiviruses were then transduced into ST cells that were approximately 50% confluent. After infection for 36 h, cells were subjected to selection with 4 μg/mL of puromycin for 7 days. Puromycin-resistant cells were identified using qRT-PCR and Western blot. For the effect of the HSP90 inhibitors, VER-82576 and KW-2478, on TGEV infection, ST cells were treated as previously described [31]. HSP90AA1 or HSP90AB1 knockdown cells, HSP90AB1 overexpression cells, and VER-82576/KW-2478-treated cells were infected with TGEV at a MOI of 0.1. At 24 hpi, TGEV M gene mRNA levels in cell suspensions and N protein levels in the cell lysis were detected by qRT-PCR and Western blot, respectively. The viral titer in the supernatant was analyzed by TCID_50_. 

### 4.4. Cell Viability Assay

The viabilities of HSP90AA1 or HSP90AB1 knockdown cells and HSP90AB1 overexpression cells were assessed using the Cell Counting Kit-8 (CCK-8) (Dalian Meilun Biotech, Dalian, China). In brief, 100 μL of 105 cells/mL were aliquoted into wells of 96-well plates and then returned to the incubator for 24 h. Meanwhile, DMEM without cells served as the blank control. After washing with PBS, 10 μL of CCK-8 reagent was added to each well for 1 h at 37 °C. Absorbance was measured at 450 nm using a microplate reader (Thermo Scientific, Waltham, MA, USA). Cell viability was calculated according to the following formula: cell viability (%) = [(OD_450_ KD/OVER-OD_450_ blank)/(OD_450_ WT-OD_450_ blank)] × 100%.

### 4.5. Effect of VER-82576 on Different Life Cycles of TGEV Infection

To further determine the effect of VER-82576 on the different life cycles of TGEV infection, a time-of-addition assay was carried out. For the pretreatment assay, ST cell monolayers in 6-well plates were pretreated with 0, 250, 500, and 1000 nM VER-82576 for 1 h at 37 °C. Then, the cells were incubated with TGEV at an MOI of 0.1 in the absence of VER-82576. After 2 h of viral adsorption, the viral supernatant was removed, and cells were further cultured with a maintenance medium without VER-82576 until 23 h after viral infection. For the cotreatment assay, ST cell monolayers in 6-well plates were infected with TGEV at an MOI of 0.1 in the presence of VER-82576 for 2 h at 37 °C. Then, the viral supernatant was removed, and the cells were further cultured with a maintenance medium without VER-82576 until 23 h after viral infection. For the post-treatment assay, ST cell monolayers in 6-well plates were incubated with TGEV for 2 h at 37 °C. Subsequently, a maintenance medium containing 1000 nM VER-82576 was added at 0, 3, 6, 12, and 18 hpi until 23 h after viral infection. For all of the above assays, samples were collected for analysis 23 h after viral infection, the relative mRNA level of the TGEV M gene was determined by qRT-PCR, the viral titer in the supernatant was analyzed by TCID_50_, and the N protein levels in the cell lysis were detected by Western blot.

### 4.6. Effect of VER-82576 on TGEV-Induced Proinflammatory Cytokine Production

To detect the production of proinflammatory cytokines IL-6, IL-12, TNF-α, CXCL10, and CXCL11 induced by TGEV, ST cell monolayers in 6-well plates were washed and infected with TGEV at an MOI of 0.1. At 24 hpi, the mRNA levels of these proinflammatory cytokines were detected by qRT-PCR. To determine the influence of VER-82576 on the production of these proinflammatory cytokines induced by TGEV infection, ST cell monolayers in 6-well plates were pretreated with 0, 250, 500, and 1000 nM VER-82576 for 1 h followed by washing with PBS three times. Then, the cells were incubated with TGEV at an MOI of 0.1 in the presence of VER-82576. After 2 h of viral adsorption, the viral supernatant was removed, and the cells were further cultured with a maintenance medium containing VER-82576 until 23 h after viral infection. Cells infected with the same dose of TGEV without inhibitor treatment were used as a control. The mRNA levels of IL-6, IL-12, TNF-α, CXCL10, and CXCL11 were detected by qRT-PCR.

### 4.7. qRT-PCR

Total RNA was extracted from TGEV-infected cells using the UNIQ-10 column TRIzol total RNA Isolation Kit (Sangon, Shanghai, China) and subjected to reverse transcription using the PrimeScript RT Reagent Kit with gDNA Eraser (Takara, Beijing, China). The cDNA was used for qRT-PCR with TB Green Premix Ex Taq II and the specific primers are shown in Table 2. Relative mRNA levels of the gene were calculated using the 2^−ΔΔCT^ method with the ACTB gene as an internal control. The qRT-PCR conditions were as follows: 95 °C for 30 s, then 40 cycles of 95 °C for 5 s, 55 °C for 30 s, and 72 °C for 30 s using the LightCycler 96 system (Roche, Mannheim, Germany). Each experiment consisted of three biological replicates, and qRT-PCR for each sample was performed in triplicate.

### 4.8. Western Blot

Cells were washed twice with cold PBS and then incubated for 15 min at 4 °C in RIPA lysis buffer with PMSF (Solarbio, Beijing, China). Lysates were centrifugated at 12,000 rpm for 5 min at 4 °C, and the supernatants were collected. Afterwards, 10 µL of supernatants were subjected to SDS-PAGE and then transferred to the PVDF membrane (Bio-Rad, Hercules, CA, USA) for 60 min at 250 mA. Membranes were blocked for 2 h at room temperature with 5% (*w*/*v*) of skim milk in TBST (0.05% Tween 20, 0.15 M NaCl, 1 mM Tris-HCI, pH 7.5) before being incubated overnight with either mouse monoclonal antibodies, TGEV N (1:200), or rabbit polyclonal antibodies, ACTB (1:10,000), HSP90AB1 (1:1000), and HSP90AA1 (1:1000). All antibodies were diluted in Primary Antibody Dilution Buffer (Beyotime, Shanghai, China). The membranes were washed four times for 4 min with TBST and then incubated for 45 min at room temperature with 1:5000 HRP-conjugated goat anti-mouse or anti-rabbit IgG. The membranes were washed again, and protein bands were visualized by the addition of ECL (Bio-Rad, USA), according to the manufacturer’s instructions, and quantified using Image J software v1.8.0.

### 4.9. TCID_50_

ST cell monolayers in 96-well plates were washed twice with a maintenance medium and then inoculated with 100 μL of a 10-fold serially diluted viral supernatant. At each dilution, there were eight technical replicates. After 2 h of viral adsorption, 150 μL of the maintenance medium was added to each well. The cytopathic effect (CPE) was observed for 4 days and was analyzed using the methods of Reed and Muench [52].

### 4.10. Statistical Analysis

All experiments were performed in triplicate. Data are shown as the mean ± standard deviation (SD). Student’s *t* test was used to measure the significant differences between the two groups. *p* values < 0.05 were considered statistically significant.

## Figures and Tables

**Table 1:** sgRNA for HSP90AA1 and HSP90AB1.

sgRNA	Sequences (5′-3′)
HSP90AA1	F: CACCGCGACGAGATGGTTTCCCTCA
	R: AAACTGAGGGAAACCATCTCGTCGC
HSP90AB1	F: CACCGAGGTCAAAAGGAGCCCGACG
	R: AAACCGTCGGGCTCCTTTTGACCTC

**Table 2:** Primer sequences used for qRT-PCR.

Genes	Sequences (5′-3′)	Size (bp)
TGEV M	F: GTGGAGAACGCTATTGTGCTAT	258
	R: AAATCGTAAGAGCCAAAACAAC	
IL-6	F: GATGCTTCCAATCTGGGTTC	218
	R: ATTTGTGGTGGGGTTAGGG	
IL12	F: AACCACCTGGACCATCTCA	211
	R: CCTCCACTGTGCTGGTTTT	
TNF-α	F: TCCTCACTCACACCATCAGC	222
	R: GCCCAGATTCAGCAAAGTCC	
CXCL10	F: AATCTACCTCTGCCATCATCTC	373
	R: AGTAGAAGCCCACGGAGTAAAG	
CXCL11	F: AACTATTCAAGGCTTCCCCAT	201
	R: ACATTTGCTTGCTTTGATTTG	
ACTB	F: CTTCCTGGGCATGGAGTCC	201
	R: GGCGCGATGATCTTGATCTTC	

## Data Availability

Not applicable.
